# Skeletal muscle metastasis from extrahepatic cholangiocarcinoma: A case report and literature review

**DOI:** 10.3389/fsurg.2022.922834

**Published:** 2022-08-09

**Authors:** Hongwei Qian, Zhikun Huang, Xuezheng Sun, Peitu Ren

**Affiliations:** ^1^ Department of Hepatobiliary and Pancreatic Surgery, Shaoxing People’s Hospital (Shaoxing Hospital, Zhejiang University School of Medicine), Shaoxing, China; ^2^ Shaoxing Key Laboratory of Minimally Invasive Abdominal Surgery and Precise Treatment of Tumor, Shaoxing, China

**Keywords:** cholangiocarcinoma, skeletal muscle metastasis, pathological, perineural invasion, case report

## Abstract

**Background:**

Cholangiocarcinoma (CCA) is a type of malignant tumor that arises from the epithelium of the bile ducts. According to anatomical location, CCA can be classified as intrahepatic (ICC), perihilar (PCC), or extrahepatic (ECC). CCA can invade and metastasize to other tissues in various ways, but distal skeletal muscle metastasis (SMM) is extremely rare. There are several reports on SMM from ICC or PCC, but SMM from ECC has not yet been reported.

**Case presentation:**

A 71-year-old woman was diagnosed with ECC, for which she underwent pancreatoduodenectomy and partial hepatectomy. Nine months after surgery, she was re-admitted to the hospital complaining of a rapidly growing mass on her right thigh with progressive lower extremity edema. Magnetic resonance imaging of the right thigh showed two masses with iso-signal intensity on T1-weighted images and hyper-intensity on T2-weighted images compared with the surrounding muscles. Pathological examination of the fine-needle biopsy specimen revealed that it was similar to the previously detected ECC, and the diagnosis was metastasis of ECC. The patient was treated with opioid analgesics and died of systemic failure three months later.

**Conclusion:**

SMM should be considered during the follow-up period despite its low incidence, and perineural invasion may be an essential pathway of distant metastasis in CCA.

## Introduction

Cholangiocarcinoma (CCA) is a type of malignant tumor that arises from the epithelium of the bile ducts. On the basis of the anatomical location, CCA can be classified as intrahepatic cholangiocarcinoma (ICC), perihilar cholangiocarcinoma (PCC), or extrahepatic cholangiocarcinoma (ECC). There has been an increase in the early detection rate of CCA because of recent improvements in imaging technology, but the treatment effects are still very poor (1, 2). ECC can invade and metastasize to other organs *via* various routes, such as lymphatic metastasis, nerve metastasis, and direct invasion. Other sites of metastasis, including local lymph nodes, liver, lungs, bones, and skeletal muscle metastasis (SMM) in the thigh are rare. Here, we report a case of ECC with short-term SMM in the right thigh.

## Case presentation

**Figure 1:**
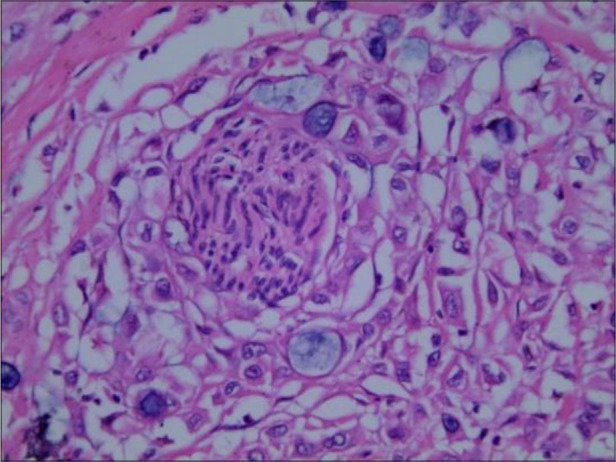
The tumor invaded nerve tissue (HE staining, 400×).

A 71-year-old woman complaining of “a rapidly growing mass on her right thigh at nine months after pancreatoduodenectomy and partial hepatectomy for ECC” was admitted to our hospital. Nine months previous, the patient was admitted to our hospital with complaints of yellow skin and sclera, yellow urine, and upper abdominal discomfort for two weeks. She underwent pancreatoduodenectomy with ECC diagnosis and received chemotherapy with tegafur after discharge. The postoperative pathological diagnosis was poorly differentiated adenocarcinoma of bile duct, involving the pancreatic tissue, cystic duct, and focal nerve; no vascular infiltration or lymph node metastasis was found (Figure 1). Before surgery, tumor marker levels were CA199 244.93 U/ml, CA50 94.16 iU/ml, and CEA 4.0 ng/ml, and those after surgery were CA199 83.44 U/ml, CA50 42.65 iU/ml, and CEA 2.47 ng/ml. Unfortunately, PET/CT was not performed before the surgery due to economic reasons, which could have provided a wider field of view and improved the accuracy of staging.

**Figure 2:**
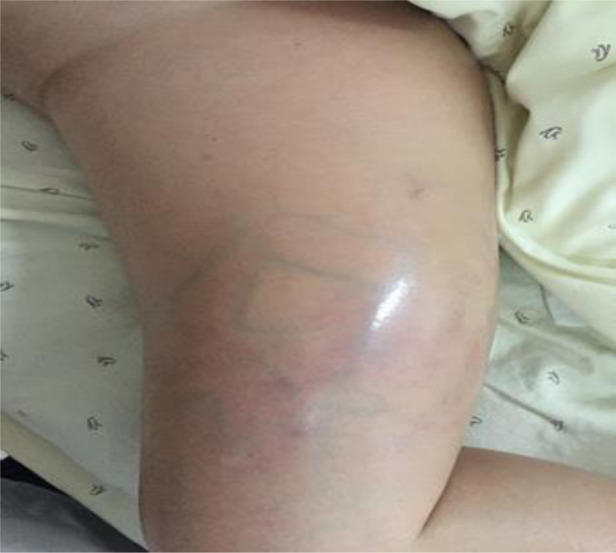
Pathological changes of swollen soft tissue in the right thigh.

**Figure 3:**
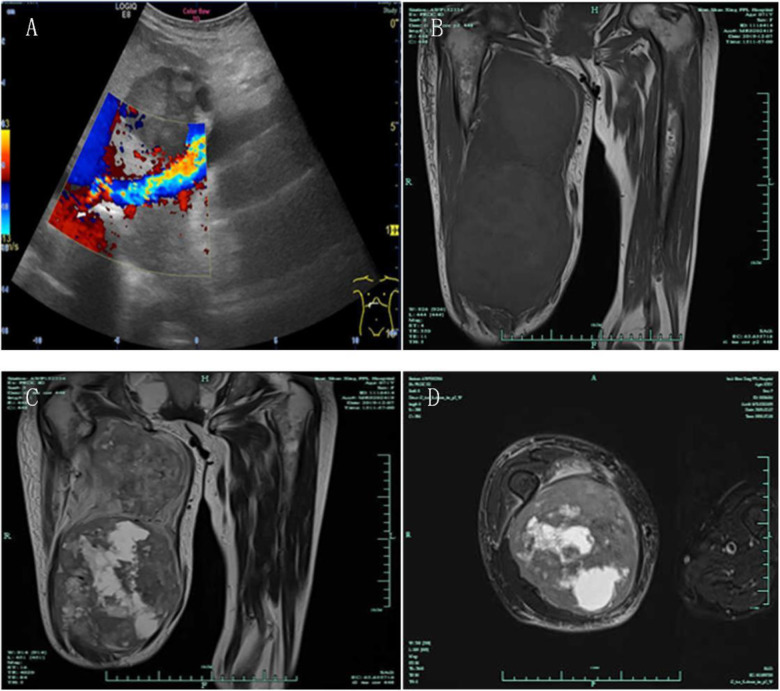
B-ultrasound showed that there was no deep vein stenosis or occlusion of the lower extremities, and the blood flow was unobstructed (**A**) MRI showed two masses with iso-signal intensity on T1-weighted images and hyper-intensity on T2-weighted images compared with the surrounding muscles ((**B**) T1 imaging, coronal plane; (**C**) T2 imaging, coronal plane; (**D**) T2 imaging, transverse plane).

She was re-admitted to the hospital because of the rapidly growing mass on her right thigh, with local pain, limited activity, and obvious swelling. The patient had retired and had no family history of hereditary disease. Physical examination: Temperature, 36.5°C; pulse, 85 bpm; respiration, 19 bpm; and blood pressure, 152/81 mmHg. The patient was emaciated but had no jaundice. There was no significant cervical, axillary, or inguinal lymphadenopathy. The heart and lungs were normal. The abdomen was flat and soft, with no tenderness or enlarged liver and spleen, and no mass was palpable. The right thigh was severely swollen, with dilated superficial veins and obvious local tenderness (Figure 2). Muscle tone, strength, and deep tendon reflexes of the limbs were normal. Laboratory examinations revealed the following: blood routine, WBC 6.2 × 10^9^/L, N 69%, Hb 6.1 g/L; liver function, ALT 13.4 U/L, AST 17.2 U/L, GGT 38.7 U/L, TBIL 9.6 µmol/L, DBIL 4.8 µmol/l; and tumor markers, CA19-9 1,184.83 U/ml, CA50 179.8 iU/ml, CA125 70.5 U/ml. B-ultrasound showed no deep vein stenosis or occlusion of the lower extremities, and the blood flow was unobstructed (Figure 3A). No new tumors were identified in the head, lungs, liver, or surrounding organs by CT examination. However, MRI examination of the right thigh showed two masses with iso-signal intensity on T1-weighted images (Figure 3B) and hyper-intensity on T2-weighted images (Figures 3C,D) compared with the surrounding muscles. The sizes of the two tumors were approximately 157 mm × 123 mm and 112 mm × 87 mm. The larger lesion with multiple irregular cystic foci oppressed the thigh muscles, which led to atrophy of the muscles.

**Figure 4:**
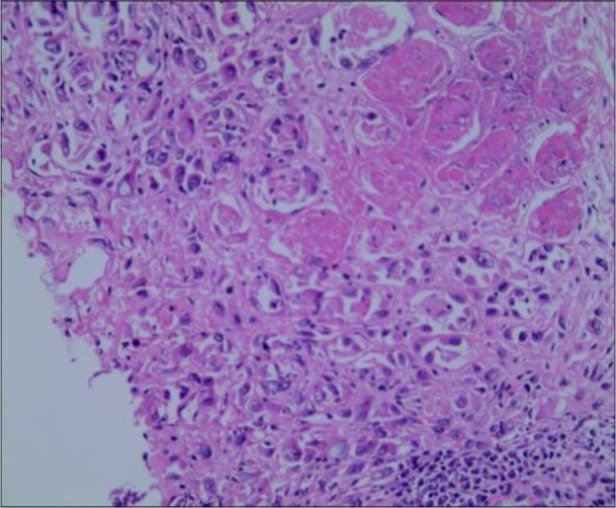
Pathological examination of the right thigh tumor showed metastatic poorly differentiated carcinoma and invasion of striated muscle (HE staining, 200×).

To further confirm the diagnosis of the lower right mass, a fine-needle biopsy was performed for pathological examinations and immunohistochemistry. Immunohistochemical staining demonstrated tumor cells with CK19(+), CKpan(+), EMA(+), Vim(−), Ki-67(+30%), CgA(−), SMA(−), DM(−), Syn(−), CD7(+), CD34(−), and CD31(−) (Figure 4), and the pathological diagnosis was postoperative metastasis of CCA. Pathological examination of the fine-needle biopsy specimen was similar to the previously detected ECC; in particular, the results of CK19(+), CK7(+), and EMA(+) confirmed the diagnosis of ECC metastasis to the right thigh.

Opioid analgesics were provided to the patient after the pathological diagnosis because she refused to receive surgical resection, systemic chemotherapy, or radiotherapy. After discharge, the patient died of systemic failure three months later.

## Discussion

CCA represents 3% of all gastrointestinal malignancies (3), and over the past few decades, both the incidence and mortality of CCA have increased worldwide(4). ECC can invade and metastasize to other tissues in various ways. Still, the incidence of SMM is very low (less than 1% of metastases of hematogenous origin), despite the skeletal muscle accounting for approximately 50% of the total body mass and its rich blood supply (5). There have been some reports on SMM from ICC or PCC, but SMM from ECC has not yet been reported. Also, in contrast to earlier findings, in which SMM was often complicated by distant metastasis such as lymphatic metastasis or liver metastasis, the patient was diagnosed with SMM from ECC with no other organ metastases were found in the head, lungs or abdominal CT examinations.

Researchers have found that the most common tumors with muscle metastases come from the pulmonary (25.1%), gastrointestinal (21.0%), and urological systems (13.2%) (6–8). SMM can appear in any striated musculature; however, most localize in the erector spine, iliopsoas, and paravertebral muscle (6, 9), but rarely in skeletal muscles of limbs. Ding et al. (10) reported a case of Hilar cholangiocarcinoma metastasizing to the rectus femoris. Nevertheless, SMM in the right thigh with a definite pathological diagnosis and obvious clinical symptoms, as described in this study, has rarely been reported.

Because of the low incidence of SMM, there are few reports in the literature (11). The reason why skeletal muscle is uninhabitable by neoplastic metastases is not well understood, but some factors have been considered to be implicated: (1) lactic acid produced by skeletal muscle (12); (2)varying tissue pressure in skeletal muscle and the influence of *β*-adrenergic receptors (13); (3)protease inhibitors located in the basement membrane (14); (4) the constant movement of skeletal muscles (5); and (5) the antitumor activity of lymphocytes or natural killer cells within the skeletal muscle (15).

The exact mechanism of SMM in this patient remains unclear, as no vascular infiltration or lymph node metastasis was found in the pathological diagnosis. And there was no significant cervical, axillary, or inguinal lymphadenopathy in the physical examination. But, focal nerve infiltration was confirmed in this case, and we suspect that perineural invasion may have an essential role in SMM besides hematogenous and lymphatic metastasis, which are considered the dominant routes for distal metastases. There is a large network of nerves in the hepatic artery, vena portae hepatica, liver interior, and extrahepatic bile duct. Feng et al. (16) thought the bile duct system is an autonomic organ controlled by a variety of nerves, and parasympathetic nervous systems contribute to malignant transformation. Perineural invasion is an important pathological feature of CCA characterized by tumor cells surrounding nerve fibers and entering the perineurium or neural fascicles. Li et al. (17) postulated that perineural invasion is a pathway of distant metastasis in CCA that has not been not widely recognized and can occur in the absence of lymphatic or vascular involvement. In our case, the perineural invasion may be a route for SMM and was associated with the poor prognosis. Despite this, there is no conclusive evidence of an association between perineural invasion and SMM, as there are no detailed pathology reports of SMM from CCA in the literature, and the results of perineural invasion were not precise (5, 10, 11, 18, 19). Therefore, additional cases with detailed pathological data are required for further research.

Because most cases of SMM are asymptomatic, non-specific, and in hidden locations (20), we speculate that under-diagnosis may also contribute to the low incidence, even though our patient was symptomatic upon presentation. Among 194 autopsies performed at the Marque de Valdecilla National Medical Center, almost 20% of patients with carcinoma had muscle metastasis (21). Therefore, SMM should be considered during the follow-up period, mainly when patients complain of progressive muscle-related symptoms. MRI plays a vital role in diagnosing SMM and is regarded as the gold standard for imaging of skeletal muscle disease (22). SMM usually has a low-intense to iso-intense signal in T1W and high signal intensity in T2W (23). In our case, the parenchymal components of the masses showed iso-intense T1W and slightly high T2W signals.

SMM is a late manifestation of malignant tumors with an extremely poor prognosis and short survival time. Treatment of SMM is controversial because there is no standard treatment regimen owing to the lack of clinical trials. Radiation therapy, chemotherapy, limited surgical management, and opioid analgesics, as in our case, are used in the treatment of this disease. Kim et al. (24) recommended surgical resection or external radiotherapy for isolated masses without other metastases. Omokawa et al. (25) reported a case of SMM from cervical cancer; they performed limited surgical procedures, and the patient was free of disease at ten months after the development of recurrent disease. However, Li et al. (19) reported a case of PCCA with SMM in which the patient died two months later after a 5-day course of 20 Gy external beam radiation in five fractions. These conflicting results suggest that additional cases with detailed clinical data are needed to interpret the molecular and pathophysiological mechanisms of SMM.

## Conclusion

Here we experienced a rare case of SMM from ECCA, which has not been previously reported. SMM should be considered during the follow-up period despite its low incidence, particularly when patients complain of progressive muscle-related symptoms.

The mechanism and treatment of SMM remain a challenge, and additional cases with detailed pathological and clinical data are required for further research. We speculate perineural invasion may have an essential role in SMM. Hence, to improve the prognosis of ECC, complete removal of the tumor with dissection of the nerve plexus around major vessels should be advocated when skeletonizing the hepatoduodenal ligament.

## Data Availability

The original contributions presented in the study are included in the article/Supplementary Material, further inquiries can be directed to the corresponding author/s.

## References

[1] Khan AS Dageforde LA. Cholangiocarcinoma. Surg Clin North Am. (2019) 99(2):315–35. 10.1016/j.suc.2018.12.004 30846037

[2] Khan SA Davidson BR Goldin R Pereira SP Rosenberg WM Taylor-Robinson SD Guidelines for the diagnosis and treatment of cholangiocarcinoma: consensus document. Gut. (2002) 51(Suppl 6):VI1–9. 10.1136/gut.51.suppl_6.vi1 12376491 PMC1867742

[3] Banales JM Cardinale V Carpino G Marzioni M Andersen JB Invernizzi P Expert consensus document: cholangiocarcinoma: current knowledge and future perspectives consensus statement from the European Network for the Study of Cholangiocarcinoma (ENS-CCA). Nat Rev Gastroenterol Hepatol. (2016) 13(5):261–80. 10.1038/nrgastro.2016.51 27095655

[4] Khan SA Tavolari S Brandi G. Cholangiocarcinoma: epidemiology and risk factors. Liver Int. (2019) 39(Suppl 1):19–31. 10.1111/liv.14095 30851228

[5] Kwon OS Jun DW Kim SH Chung MY Kim NI Song MH Distant skeletal muscle metastasis from intrahepatic cholangiocarcinoma presenting as Budd-Chiari syndrome. World J Gastroenterol. (2007) 13(22):3141–3. 10.3748/wjg.v13.i22.3141 17589935 PMC4172626

[6] Surov A Köhler J Wienke A Gufler H Bach AG Schramm D Muscle metastases: comparison of features in different primary tumours. Cancer Imaging. (2014) 14:21. 10.1186/1470-7330-14-21 25608474 PMC4331826

[7] Leitner J Pelster S Schöpf V Berghoff AS Woitek R Asenbaum U High correlation of temporal muscle thickness with lumbar skeletal muscle cross-sectional area in patients with brain metastases. PLoS One. (2018) 13(11):e0207849. 10.1371/journal.pone.0207849 30496307 PMC6264824

[8] Dohzono S Sasaoka R Takamatsu K Hoshino M Nakamura H. Low paravertebral muscle mass in patients with bone metastases from lung cancer is associated with poor prognosis. Support Care Cancer. (2020) 28(1):389–94. 10.1007/s00520-019-04843-9 31055666

[9] Carey K Bestic J Attia S Cortese C Jain M. Diffuse skeletal muscle metastases from sacral chordoma. Skeletal Radiol. (2014) 43(7):985–9. 10.1007/s00256-013-1794-1 24407557

[10] Ding G Yang J Cheng S Gong H Liu K Dai B Hilar cholangiocarcinoma with synchronous metastases to breast and skeletal muscle: a case report and literature review. Chinese German J Clin Oncol. (2006) 5(3):216–8. 10.1007/s10330-006-0467-0

[11] Lee J Lee SW Han SY Baek YH Kim SY Rhyou HI. Rapidly aggravated skeletal muscle metastases from an intrahepatic cholangiocarcinoma. World J Gastroenterol. (2015) 21(6):1989–93. 10.3748/wjg.v21.i6.1989 25684968 PMC4323479

[12] Seely S. Possible reasons for the high resistance of muscle to cancer. Med Hypotheses. (1980) 6(2):133–7. 10.1016/0306-9877(80)90079-1 7393016

[13] Yoshimura Y Isobe K Koike T Arai H Aoki K Kato H. Metastatic carcinoma to subcutaneous tissue and skeletal muscle: clinicopathological features in 11 cases. Jpn J Clin Oncol. (2011) 41(3):358–64. 10.1093/jjco/hyq199 21051532

[14] Pauli BU Schwartz DE Thonar EJ Kuettner KE. Tumor invasion and host extracellular matrix. Cancer Metastasis Rev. (1983) 2(2):129–52. 10.1007/BF00048966 6352011

[15] Sudo A Ogihara Y Shiokawa Y Fujinami S Sekiguchi S. Intramuscular metastasis of carcinoma. Clin Orthop Relat Res. (1993) 296:213–7. 10.1097/00003086-199311000-00036 8222429

[16] Feng Y-J Zhang B-Y Yao R-Y Lu Y. Muscarinic acetylcholine receptor M3 in proliferation and perineural invasion of cholangiocarcinoma cells. Hepatobiliary Pancreat Dis Int. (2012) 11(4):418–23. 10.1016/S1499-3872(12)60201-X 22893470

[17] Li CG Huang ZQ Wei LX Zhao ZM Hu MG Xiao Y [Perineural invasion in hilar cholangiocarcinoma and distribution of nerve plexuses around porta hepatis]. Zhonghua Yi Xue Za Zhi. (2012) 92(38):2699–702. 10.3760/cma.j.issn.0376-2491.2012.38.010 23290109

[18] Park SK Kim YS Kim SG Jang JY Moon JH Lee MS Detection of distant metastasis to skeletal muscle by 18F-FDG-PET in a case of intrahepatic cholangiocarcinoma. Korean J Hepatol. (2010) 16(3):325–8. 10.3350/kjhep.2010.16.3.325 20924217 PMC3304591

[19] Li J Henry MR Roberts LR. Rare distant skeletal muscle metastasis from hilar cholangiocarcinoma: report of a case. J Gastrointest Cancer. (2011) 42(3):171–3. 10.1007/s12029-010-9237-x 21153451

[20] Cione GP Arciero G De Angelis CP Marano A Farella N Cerrone C [Intrahepatic cholangiocarcinoma: case report]. Suppl Tumori. (2005) 4(3):S46–47. 16437896

[21] Acinas Garcia O Fernandez FA Satue EG Buelta L Val-Bernal JF. Metastasis of malignant neoplasms to skeletal muscle. Rev Esp Oncol. (1984) 31(1):57–67. 6545428

[22] Pretorius ES Fishman EK. Helical CT of skeletal muscle metastases from primary carcinomas. AJR Am J Roentgenol. (2000) 174(2):401–4. 10.2214/ajr.174.2.1740401 10658714

[23] Suto Y Yamaguchi Y Sugihara S. Skeletal muscle metastasis from lung carcinoma: MR findings. J Comput Assist Tomogr. (1997) 21(2):304–5. 10.1097/00004728-199703000-00027 9071306

[24] Kim YW Seo KJ Lee SL Kwon KW Hur J An HJ Skeletal muscle metastases from breast cancer: two case reports. J Breast Cancer. (2013) 16(1):117–21. 10.4048/jbc.2013.16.1.117 23593092 PMC3625758

[25] Omokawa N Mabuchi S Iwai K Kawahara N Kawaguchi R Sugimoto S Skeletal muscle metastasis as a first site of recurrence of cervical cancer: a case report and review of the literature. Medicine (Baltimore). (2020) 99(19):e20056. 10.1097/MD.0000000000020056 32384470 PMC7220549

